# IL4Rα and IL13Rα1 Are Involved in the Development of Human Gallbladder Cancer

**DOI:** 10.3390/jpm12020249

**Published:** 2022-02-09

**Authors:** Sung Woo Ahn, Chang Min Lee, Mi-Ae Kang, Usama Khamis Hussein, Ho Sung Park, Ae-Ri Ahn, Hee Chul Yu, Jae Do Yang, Yung-Hun Yang, Kyungmoon Park, Jongsung Lee, Kyu Yun Jang, See-Hyoung Park

**Affiliations:** 1 Department of Surgery, Jeonbuk National University Medical School, Jeonju 54896, Korea; surgeonahn@gmail.com (S.W.A.); hcyu@jbnu.ac.kr (H.C.Y.); hirojawa@jbnu.ac.kr (J.D.Y.); 2 Department of Bio and Chemical Engineering, Hongik University, Sejong 30016, Korea; yycc456@naver.com (C.M.L.); pkm2510@hongik.ac.kr (K.P.); 3 Zoonolab Inc., Seoul 04799, Korea; makang53@hanmail.net; 4 Department of Pathology, Jeonbuk National University Medical School, Jeonju 54896, Korea; usamahussein@jbnu.ac.kr (U.K.H.); hspark@jbnu.ac.kr (H.S.P.); xoxoyool@naver.com (A.-R.A.); 5 Research Institute of Clinical Medicine of Jeonbuk National University, Jeonju 54896, Korea; 6 Biomedical Research Institute of Jeonbuk National University Hospital, Jeonju 54896, Korea; 7 Department of Biological Engineering, Konkuk University, Seoul 05029, Korea; seokor@konkuk.ac.kr; 8 Department of Integrative Biotechnology, Sungkyunkwan University, Suwon 03063, Korea

**Keywords:** IL4Rα, IL13Rα1, JAK2, AZD1480, gallbladder cancer

## Abstract

Background: Gallbladder cancer is commonly associated with inflammation, which indicates that inflammation-related cytokines and cytokine receptors are related to the progression of gallbladder cancers. Interleukin 4 (IL4) is a well-known cytokine that promotes the differentiation of naive helper T cells (Th0) to T helper type 2 cells (Th2). IL13 is a cytokine that is secreted by Th2 cells. IL4 and IL13 are closely related in immune responses. However, the role of IL4Rα and IL13Rα1 signaling pathway has not been fully understood in the development of gallbladder cancer. Methods: In human gallbladder carcinomas, the expression of IL4Rα and IL13Rα1 were evaluated with immunohistochemical staining in tissue microarray tissue sections. After knockdown of IL4Rα or IL13Rα1, cell assays to measure the proliferation and apoptosis and Western blotting analysis were conducted in SNU308 human gallbladder cancer cells. Since Janus kinases2 (JAK2) was considered as one of the down-stream kinases under IL4Rα and IL13Rα1 complex, the same kinds of experiments were performed in SNU308 cells treated with AZD1480, Janus-associated kinases2 (JAK2) inhibitor, to demonstrate the cytotoxic effect of AZD1480 in SNU308 cells. Results: Immunohistochemical expression of IL4Rα was significantly associated with the expression of IL13Rα1 in human carcinoma tissue. In univariate analysis, nuclear expression of IL4Rα, cytoplasmic expression of IL4Rα, nuclear expression of IL13Rα1, and cytoplasmic expression of IL13Rα1 were significantly associated with shorter overall survival and shorter relapse-free survival. Multivariate analysis revealed nuclear expression of IL4Rα as an independent poor prognostic indicator of overall survival and relapse-free survival. Then, we found that knockdown of IL4Rα or IL13Rα1 decreased viability and induced apoptosis in SNU308 cells via activation of FOXO3 and similarly, AZD1480 decreased viability and induced apoptosis in SNU308 cells with dose dependent manner. Conclusions: Taken together, our results suggest that IL4Rα and IL13Rα1 might be involved in the development of human gallbladder cancer cells and IL4Rα and IL13Rα1 complex/JAK2 signaling pathway could be efficient therapeutic targets for gallbladder cancer treatment.

## 1. Introduction

The gallbladder is a small organ that is located below the right lobe of the liver which stores and secretes bile to help digest fats [1]. Gallbladder cancer is not as common as other cancers, however, certain Asian countries such as Korea and Japan showed high incidence rates [2]. According to the recent statistics, even though the size of gallbladder cancer is smaller than 2 cm, this cancer can cause approximately 170,000 deaths annually, which accounts for 1.7% of deaths caused by all cancers [3]. Because gallbladder cancer is difficult to detect in early state of cancer and is not sensitive to chemotherapy, prognosis is even poorer than other cancers [4,5]. Surgical resection has been considered only potential therapeutic method; however, efficacy is still controversial because gallbladder and a part of the cystic duct should be removed, which causes serious problems in digestion system [6]. Therefore, alternative therapeutic methods that have fewer side effects and exert strong therapeutic effects need to be developed. 

Interleukin 4 (IL4) and 13 (IL13) are typical cytokines that play crucial roles in the inflammatory response caused by lot of antigens such as parasite or allergen [7]. IL4 is secreted by T helper type 2 (Th2) cells and mast cells [8,9]. IL4 has many biological functions in immune system such as stimulation B/T cell and the differentiation of B cells [10,11]. IL13 is secreted by various immune cells such as Th2 cells, CD4 cells, and mast cells [12]. IL13 also has many biological roles including regulation of IgE synthesis, mucus hypersecretion, and airway hyperresponsiveness [13,14]. Therefore, these cytokines are key targets for treatment of immune diseases such as inflammation, atopic dermatitis, and asthma [15,16,17]. It has been reported that receptors for IL4 and IL13 (IL4Rα or IL13Rα1) in several type of cancers is associated with poor prognosis and thus could be therapeutic targets of cancer treatment [18]. In line with this study, the role of the expression of IL4Rα and IL13Rα1 as prognostic indicator has been reported in various human cancers. The poor prognosis of the patients having a tumor with higher expression of IL4Rα has been reported in renal cell carcinoma [19], malignant mesothelioma [20], and soft tissue sarcomas [21]. Higher expression of IL13Rα1 was also presented as an indicator of poor prognosis of breast cancer [22], renal cell carcinoma [19], soft-tissue sarcomas [21], and glioblastoma [23]. Therefore, the result of this study suggests the expressions of IL4Rα and IL13Rα1 as novel prognostic indicators of gallbladder carcinoma patients. However, the exact effects of IL4Rα or IL13Rα1 in gallbladder cancer development have not been studied.

Janus kinase 2 (JAK2), known as non-receptor protein tyrosine kinase, is a member of Janus kinase family [24]. JAK2 is related to cytokine receptors and plays a pivotal role in signal transduction by regulating tyrosine phosphorylation [25]. JAK2 is a key factor of cell proliferation and division and is especially one of key kinases for regulating production of blood cells in bone marrow [26,27]. Interestingly, JAK2 mutation is found in most of patients with myeloproliferative cancer and polycythemia vera [28]. This suggested that the JAK2 signaling pathway could be an important target in cancer treatment. In this study, we investigate the cytotoxic effect of AZD1480 that is a JAK2 inhibitor in SNU308 human gallbladder cancer cells. Furthermore, since JAK2 was considered as one of the down-stream kinases under IL4Rα and IL13Rα1 complex [19], we tried to investigate the effect of IL4Rα or IL13Rα1 signaling pathway in SNU308 cell.

## 2. Materials and Methods

### 2.1. Human Gallbladder Carcinomas

To evaluate the clinicopathological significance of the expression of IL4Rα and IL13Rα1 in human gallbladder cancers, 122 cases of gallbladder carcinomas treated between January 2000 and December 2019 were evaluated in this study. The information on the clinicopathological factors was obtained by reviewing the medical record and original histologic microscopic slides. The cases included in this study had a complete medical record, histologic slides, and tissue blocks. Then, all cases were classified according to the latest WHO classification of gallbladder tumor and staged according to the latest American Joint Committee Cancer Staging manual. The factors evaluated in this study were age, sex, preoperative serum level of CEA and CA19-9, tumor stage, T category, lymph node metastasis, distant metastasis, histologic type, and histologic grade of cancer. This study was performed with the approval of the institutional review board of Jeonbuk National University Hospital (IRB number, CUH 2020-10-026-002). In this approval, written informed consent was waived because of the anonymous and retrospective nature of this study.

### 2.2. Immunohistochemical Staining and Scoring

To evaluate the expression of IL4Rα and IL13Rα1 in gallbladder cancer tissue, tissue microarray (TMA) with 3.0 mm core was constructed. Two TMA cores were constructed in each case of gallbladder cancer from the area mainly composed of tumor cells with the highest histologic grade. The tissue sections derived from the TMA tissue blocks were used in immunohistochemical staining. The tissue sections were deparaffinized and boiled for 20 min in a pH 6.0 antigen retrieval solution (DAKO, Glostrup, Denmark) using a microwave oven. Thereafter, the tissue sections were incubated with primary antibodies for IL4Rα (1:100, Santa Cruz Biotechnology, Santa Cruz, CA, USA) and IL13Rα1 (1:100, Santa Cruz Biotechnology) overnight at 4 °C. The incubated slides were incubated with secondary antibodies and visualized using the DAKO Envision system (DAKO). Then, based on the previous report of the expression pattern of IL4Rα and IL13Rα1 in human cancer tissue, the expression of IL4Rα and IL13Rα1 were separately analyzed according to their nuclear and cytoplasmic expression. Immunohistochemical expression of IL4Rα and IL13Rα1 was scored by adding the staining intensity score (score 0; no staining, score 1; weak staining, score 2; intermediate staining, score 3; strong staining) and staining area score (score 0; 0%, score 1; 1%, score 2; 2–10%, score 3; 11–33%, score 4; 34–66%, score 5; 67–100%) [21,29]. Therefore, the immunohistochemical staining score in one TMA core was ranged from zero to eight. Because two TMA cores were established in each case, the final immunohistochemical staining score was obtained by adding the scores of the first core and second core. Therefore, the final score ranged from 0 to 16.

### 2.3. Reagents

AZD1480 was from MedChemExpress (Monmouth Junction, NJ, USA) and was dissolved in dimethyl sulfoxide (Sigma, St. Louis, MO, USA). The stock solution (40 mM) was stored at −20 °C. The 35~40% formaldehyde and Triton X-100 were from Sigma. Muse Cell Analyzer was from Merck Millipore (Billerica, MA, USA). FlowJo software to analyze raw data from Muse Cell Analyzer was from FlowJo LCC (version 10.5.3, Ashland, OR, USA). Primary antibodies in this study were as follows: anti-β-actin, anti-GAPDH, anti-Lamin A/C, anti-IL4Rα, and anti-IL13Rα1 were from Santa Cruz Biotechnology, and anti-cleaved PARP-1, anti-cleaved caspase-3, anti-FOXO3, anti-p27, anti-Bax, anti-Bcl-2, anti-JAK2, and anti-pJAK2 were from Cell Signaling Technology (Danvers, MA, USA). Horseradish peroxidase (HRP)-conjugated anti-rabbit and anti-mouse secondary antibodies were from Santa Cruz Biotechnology. 

### 2.4. Cell Culture

SNU308 cells were purchased from Korean Cell line Bank (Seoul, Korea). Cells were maintained in RPMI1640 medium (Hyclone, Logan, UT, USA) supplemented with 10% heat-inactivated fetal bovine serum (FBS, Sigma) and 1% penicillin/streptomycin (Sigma). Cells were incubated in humidified incubator (37 °C, 5% CO_2_). 

### 2.5. Transfection

SNU308 cells were plated in 60 mm dishes (3 × 10^5^ cells/dish) and incubated overnight. Then, 0.6 mL of warm serum free media was added to a 1.7 mL tube. 5 uL of control, IL4Rα, or IL13Rα1 siRNA (Santa Cruz, the concentration of siRNA stock solution is 10 uM) and 15 µL of lipofectamine 2000 (Waltham, MA, USA) were added to media respectively and incubated for 15 min. After incubation, the media containing siRNA and the media containing lipofectamine 2000 were mixed and incubated for 15 min. After 15 min of incubation, cells were washed twice with PBS and the siRNA and lipofectamine 2000 mixtures were added to cells. Cells were incubated at 37 °C in incubator for 6 h. After 6 h of incubation, the media was removed and fresh media containing FBS and antibiotics was added to cells.

### 2.6. Water-Soluble Tetrazolium Salt-1 (WST-1) Assay

SNU308 cells were plated in 96-well plates (3 × 10^3^ cells/well) and incubated overnight. After incubation, cells were transfected with control, IL4Rα, or IL13Rα1 siRNA or treated with various concentrations of AZD1480 (0, 5, 10, and 20 µM) for 24, 48, and 72 h. Then, media was replaced with 100 µL of fresh media containing 10% EZ-Cytox (DoGenBio, Seoul, Korea) and the plates were incubated for 1 h in 37 °C incubator. The absorbance at 450 nm was measured by spectrophotometer (Molecular Devices, Mountain View, CA, USA).

### 2.7. Morphological Change Analysis

SNU308 cells were plated in 60 mm dishes (3 × 10^5^ cells/dish) and incubated overnight. After incubation, cells were transfected with control, IL4Rα, or IL13Rα1 siRNA or treated with AZD1480 (0, 5, 10, and 20 µM) for 24, 48, and 72 h. Then, cells were observed at an interval of 24 h by microscopy (CKX53, Olympus, Tokyo, Japan). 

### 2.8. Cell Counting Assay

SNU308 cells were plated in 6-well plates (2 × 10^4^ cells/well) and incubated overnight. After incubation, cells were transfected with control, IL4Rα, or IL13Rα1 siRNA or treated with AZD1480 (0, 10, and 20 µM) for 24, 48, and 72 h. Then, cells were washed with PBS twice, detached by Trypsin-EDTA, and centrifuged (1350 rpm, 3 min). After removing supernatant, cells were suspended with 1 mL of media and the number of cells was counted using hemocytometer. 

### 2.9. Colony Formation Assay

SNU308 cells were plated in 6-well plate (1 × 10^3^ cells/well) and incubated overnight. After incubation, cells were transfected with control, IL4Rα, or IL13Rα1 siRNA or treated with AZD1480 (0, 10, and 20 µM) for 2 weeks and culture media was changed with the fresh media containing 10% FBS every two day. Then, cells were washed with PBS twice, fixed by 3.5~4% formaldehyde in PBS, and stained with 1% crystal violet solution (Sigma). After stained, the number of colonies was counted.

### 2.10. Annexin V Staining Analysis

SNU308 cells were plated in 6-well plates (2 × 10^5^ cells/well) and incubated overnight. After incubation, cells were transfected with control, IL4Rα, or IL13Rα1 siRNA or treated with AZD1480 (0, 5, 10, and 20 µM) for 48 h. Then, apoptotic cell population was analyzed by Muse Cell Analyzer and Muse Annexin V and Dead Cell Kit (Merck Millipore) according to manufacturer’s protocol. 

### 2.11. Caspase-3/7 Activity Assay

SNU308 cells were plated in 6-well plates (2 × 10^5^ cells/well) and incubated overnight. After incubation, cells were transfected with control, IL4Rα, or IL13Rα1 siRNA or treated with AZD1480 (0, 5, 10, and 20 µM) for 48 h. Then, caspase-3 and -7 activities were measured by Muse Cell Analyzer and Muse Caspase-3/7 Kit (Merck Millipore) according to manufacturer’s protocol. 

### 2.12. Mitochondria Depolarization Assay

SNU308 cells were plated in 6-well plates (2 × 10^5^ cells/well) and incubated overnight. After incubation, cells were transfected with control, IL4Rα, or IL13Rα1 siRNA or treated with AZD1480 (0, 5, 10, and 20 µM) for 48 h. Then, depolarization of mitochondria was detected by Muse Cell Analyzer and Muse Mito-Potential Kit (Merck Millipore) according to manufacturer’s protocol.

### 2.13. Cell Cycle Analysis

SNU308 cells were plated in 6-well plates (2 × 10^5^ cells/well) and incubated overnight. After incubation, cells were transfected with control, IL4Rα, or IL13Rα1 siRNA or treated with AZD1480 (0, 5, 10, and 20 µM) for 48 h. Then, population of each cell cycle status was analyzed by Muse Cell Analyzer and Muse Cell Cycle Kit (Merck Millipore) according to manufacturer’s protocol. 

### 2.14. Terminal Deoxynucleotidyl Transferase dUTP Nick End Labeling (TUNEL) Assay

SNU308 cells were plated in 6-well plate (2 × 10^5^ cells/well) and incubated overnight. After incubation, cells were transfected with control, IL4Rα, or IL13Rα1 siRNA or treated with AZD1480 (0, 5, 10, and 20 µM) for 48 h. Then, cells were washed with PBS twice and fixed with 3.5~4% formaldehyde in PBS. After fixation, TUNEL assay was performed according to manufacturer’s protocol (TUNEL detection system, Promega, Madison, WI, USA). DNA damage of cell was observed by fluorescence microscopy (CKX53, Olympus). 

### 2.15. Comet Assay

SNU308 cells were plated in 6-well plates (1 × 10^5^ cells/well) and incubated overnight. After incubation, cells were transfected with control, IL4Rα, or IL13Rα1 siRNA or treated with AZD1480 (0, 5, 10, and 20 µM) for 48 h. Then, comet assay was performed according to manufacturer’s protocol (Abcam, Cambridge, MA, USA). Comet tail of cell was observed by fluorescence microscopy (CKX53, Olympus).

### 2.16. Separation of Cytosol/Nuclear Proteins

SNU308 cells were plated in 100 mm dishes (2 × 10^6^ cells/dish) and incubated overnight. After incubation, cells were transfected with control, IL4Rα, or IL13Rα1 siRNA or treated with AZD1480 (0, 5, 10, and 20 µM) for 48 h. Then, cells were harvested with scraper and centrifuged. Cytosol and nuclear proteins were isolated respectively by Nuclear/Cytosol Fractionation Kit (BioVision, Milpitas, CA, USA) according to manufacturer’s protocol. 

### 2.17. Western Blotting

SNU308 cells were plated in 6-well culture plates (2 × 10^5^ cells/well) and incubated overnight. After incubation, cells were transfected with control, IL4Rα, or IL13Rα1 siRNA or treated with AZD1480 (0, 5, 10, and 20 µM) for 48 h. Then, cells were harvested with scraper centrifuged, and lysed with 1 × RIPA buffer (Cell Signaling Technology). Protein was separated on 10–12% sodium dodecyl sulfate (SDS)-polyacrylamide gels and transferred onto 0.45 µm PVDF Immobilon-P membrane (Merch Millipore). The membranes were blocked with 5% bovine serum albumin (BSA, Sigma), incubated with the primary antibodies (1:1000 dilution), and incubated with the secondary antibody (1:10,000 dilution). Bands were detected using ECL (GE Healthcare, Chicago, IL, USA) and Chemi-doc detection system (Bio-Rad, Hercules, CA, USA). 

### 2.18. Statistical Analysis

Data was analyzed with SPSS software (version 20.0, Chicago, IL, USA). Two-sided Student’s *t*-test was conducted and *p*-value lower than 0.05 was considered to have statistical significance. 

## 3. Results

### 3.1. Expression of IL4Rα and IL13Rα1 in Gallbladder Carcinomas and Their Association with Clinicopathologic Variables

**Figure 1:**
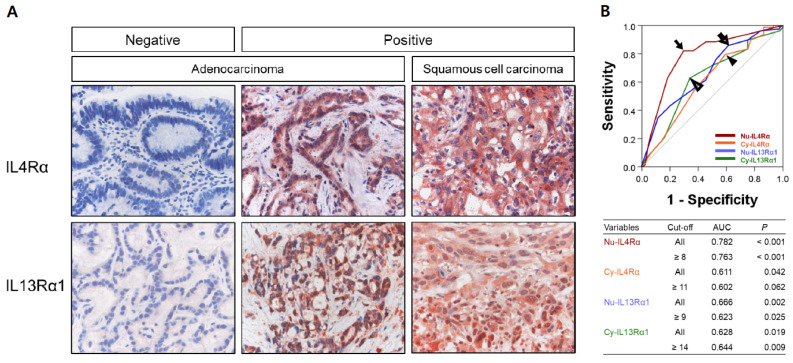
Immunohistochemical expression of IL4Rα and IL13Rα1 in human gallbladder carcinoma tissue. (**A**) The nuclear and cytoplasmic expression of IL4Rα and IL13Rα1 are seen in positive examples of adenocarcinoma and squamous cell carcinoma components of gallbladder carcinomas. Original magnification: x400. (**B**) Statistical analysis to determine immunohistochemical positivity of the expression of IL4Rα and IL13Rα1. The cut-off points are determined by using receiver operating characteristic curve analysis at the points with the highest area under the curve (AUC) to predict cancer related death of patients. The cut-off points for the expression of nuclear IL4Rα (Nu-IL4Rα, black arrow), cytoplasmic IL4Rα (Cy-IL4Rα, arrowhead), nuclear IL13Rα1 (Nu-IL13Rα1, empty arrow), and cytoplasmic IL13Rα1 (Cy-IL13Rα1, empty arrowhead) are indicated in the receiver operating characteristic curve.

Immunohistochemically, IL4Rα and IL13Rα1 were expressed in cytoplasm and nuclei of gallbladder cancer cells (Figure 1). The examples of negative and positive expressions of IL4Rα and IL13Rα1 in adenocarcinoma and squamous cell carcinoma components of gallbladder carcinoma are shown in Figure 1A. The cut-off points for the immunohistochemical staining scores for the nuclear expression of IL4Rα (Nu-IL4Rα), cytoplasmic expression of IL4Rα (Cy-IL4Rα), nuclear expression of IL13Rα1 (Nu-IL13Rα1), and cytoplasmic expression of IL13Rα1 (Cy-IL13Rα1) were determined by ROC curve analysis (Figure 1B). The cut-off points have the highest area under the curve (AUC) to predict cancer-related death of patients, and the cut-off points for Nu-IL4Rα, Cy-IL4Rα, Nu-IL13Rα1, and Cy-IL13Rα1 were 8, 11, 9, and 14, respectively (Figure 1B). In these cut-off values, Nu-IL4Rα positivity was significantly associated with age of the patients (*p* = 0.010), tumor stage (*p* = 0.040), T category of the stage (*p* = 0.035), distant metastasis (*p* = 0.025), histologic grade (*p* = 0.012), and the expression of Cy-IL4Rα (*p* < 0.001), Nu-IL13Rα1 (*p* = 0.011), and Cy-IL13Rα1 (*p* = 0.004) (Table 1). Cy-IL4Rα-positivity was significantly associated with age of the patients (*p* = 0.003) and sex (*p* = 0.011) (Table 1). Nu-IL13Rα1-positivity was significantly associated with sex (*p* = 0.004), and Cy-IL13Rα1-positivity was significantly associated with histologic grade (*p* = 0.008) (Table 1). 

### 3.2. Prognostic Significance of the Expressions of IL4Rα and IL13Rα1 in Gallbladder Carcinomas

**Figure 2:**
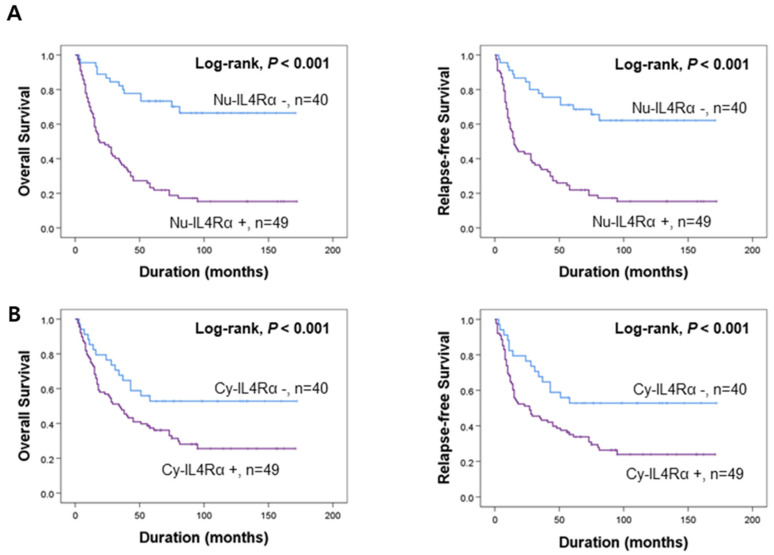

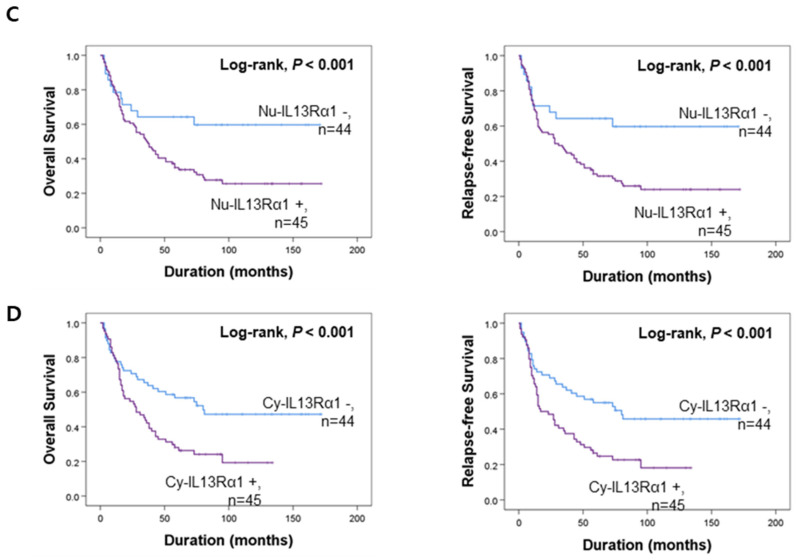
Survival analysis according to the expression of IL4Rα and IL13Rα1 in gallbladder carcinoma patients. Kaplan–Meier survival curves of overall survival and relapse-free survival according to the expression of nuclear IL4Rα (Nu-IL4Rα) (**A**), cytoplasmic IL4Rα (Cy-cIL4Rα) (**B**), nuclear IL13Rα1 (Nu-IL13Rα1) (**C**), and cytoplasmic IL13Rα1 (Cy-IL13Rα1) (**D**).

The potential prognostic clinicopathologic factors and the expressions of Nu-IL4Rα, Cy-IL4Rα, Nu-IL13Rα1, and Cy-IL13Rα1 were evaluated for their impact on OS and RFS of gallbladder carcinoma patients. In univariate analysis, the factors associated with both cancer-specific survival (CSS) and relapse-free survival (RFS) were examined (age of the patients (OS; *p* < 0.001, RFS; *p* < 0.001), serum level of CA19-9 (OS; *p* = 0.020, RFS; *p* = 0.031), tumor stage (OS; *p* < 0.001, RFS; *p* < 0.001), T category of the stage (OS; *p* < 0.001, RFS; *p* < 0.001), lymph node metastasis (OS; *p* = 0.006, RFS; *p* = 0.012), distant metastasis (OS; *p* < 0.001, RFS; *p* < 0.001), histologic type of cancer (OS; *p* = 0.002, RFS; *p* = 0.005), histologic grade (OS; *p* < 0.001, RFS; *p* < 0.001), the expression of Nu-IL4Rα (OS; *p* < 0.001, RFS; *p* < 0.001), Cy-IL4Rα (OS; *p* = 0.023, RFS; *p* = 0.011), Nu-IL13Rα1 (OS; *p* = 0.016, RFS; *p* = 0.010), and Cy-IL13Rα1 (OS; *p* = 0.004, RFS; *p* = 0.003) (Table 2). The carcinoma positive for Nu-IL4Rα had a 4.614-fold (95% CI (95% confidential interval); 2.575–8.269) greater risk of death and a 4.019-fold (95% CI; 2.311–6.988) greater risk of relapse or death compared with carcinoma negative for Nu-IL4Rα (Table 2). The Cy-IL4Rα-positivity predicted a 1.895-fold (95% CI; 1.092–3.288) greater risk of death and a 2.048-fold (95% CI; 1.183–3.548) greater risk of relapse or death of gallbladder carcinoma patients (Table 2). The positivity for Nu-IL13Rα1 was associated with a 2.197-fold (95% CI; 1.159–4.168) greater risk of death and a 2.310-fold (95% CI; 1.220–4.376) greater risk of relapse or death of patients (Table 2). The Cy-IL13Rα1-positivity predicted a 1.987-fold (95% CI; 1.250–3.161) greater risk of death and a 2.008-fold (95% CI; 1.271–3.172) greater risk of relapse or death of patients (Table 2). The results of Kaplan–Meier survival analysis were presented in Figure 2 and the expressions of Nu-IL4Rα, Cy-IL4Rα, Nu-IL13Rα1, and Cy-IL13Rα1 were significantly associated with OS and RFS. Multivariate survival analysis was performed by including the factors significantly associated with OS or RFS. Age of patients, serum level of CA19-9, tumor stage, T category, lymph node metastasis, distant metastasis, histologic type, histologic grade, Nu-IL4Rα, Cy-IL4Rα, Nu-IL13Rα1, and Cy-IL13Rα1 were included in multivariate analysis. In multivariate analysis age of the patients (OS; *p* < 0.001, RFS; *p* < 0.001), tumor stage (OS; *p* = 0.010, RFS; *p* = 0.043), T category (OS; *p* = 0.048, RFS; *p* = 0.016), and Nu-IL4Rα expression (OS; *p* < 0.001, RFS; *p* < 0.001) were independent indicators of OS and RFS (Table 3). Especially, the gallbladder carcinoma patients Nu-IL4Rα-positive tumors had a 3.379-fold (95% CI; 1.825–6.254) greater risk in OS analysis and a 2.919-fold (95% CI; 1.622–5.253) greater risk in RFS analysis compared with the patients with negative for Nu-IL4Rα (Table 3). Taken together, in human gallbladder carcinoma tissue, we found a statistically significant association between the expression IL4R and IL13Rα1 and poor prognostic properties by analyzing tissue microarray.

### 3.3. Knockdown of IL4Rα or IL13Rα1 Displays Anti-Proliferative Activity in SNU308 Cells

**Figure 3:**
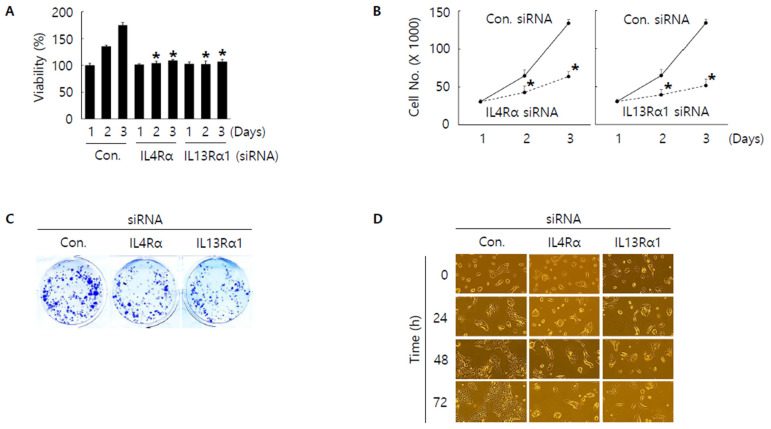
Anti-proliferative effect by knockdown of IL4Rα or IL13Rα1 in SNU308 cells. (**A**) Cell viability (WST-1 assay) was measured for 24, 48, and 72 h after transfection of siRNA. The percentage of SNU308 cell viability transfected with siRNAs against IL4Rα and IL13Rα1 was compared with the cells transfected with control siRNA (cell viability = 100%) at 24 h after transfection. (**B**) The number of viable cells was counted with hemocytometer for 24, 48, and 72 h after transfection of siRNA. (**C**) After transfection of siRNA, cells were grown for 14 days in fresh media containing 10% FBS. Crystal violet staining was performed to visualize colonies. (**D**) Cell morphological image was captured by microscopy for 24, 48, and 72 h after transfection of siRNA. * *p* < 0.05; asterisks mean a significant difference between control siRNA group and IL4Rα or IL13Rα1 siRNA group. Results are mean ± SD of at least three independent experiments.

To investigate the role of IL4Rα and IL13Rα1 in SNU308 cells, IL4Rα and IL13Rα1 were knocked-down through siRNA. First, we evaluated the anti-proliferative effect. WST-1 assay results indicated that the amount of live SNU308 cells was reduced after knockdown of IL4Rα or IL13Rα1 (Figure 3A). IL4Rα or IL13Rα1 knockdown groups showed significant decrease of cell numbers compared to control group (Figure 3B). There is no significant difference between IL4Rα and IL13Rα1 groups. These results were confirmed by colony formation assay (Figure 3C). Additionally, morphological change analysis showed that the growth of cells was not observed in SNU308 cells with knockdown of IL4Rα or IL13Rα1 but the uncontrolled growth of cells was increased in the control group with time-dependent manner (Figure 3D). Overall, our results indicated that knockdown of IL4Rα or IL13Rα1 by siRNA transfection displayed anti-proliferative activity in SNU308 cells.

### 3.4. Knockdown of IL4Rα or IL13Rα1 Induces Apoptosis in SNU308 Cells via Regulation of JAK2/FOXO3 Pathways

**Figure 4:**
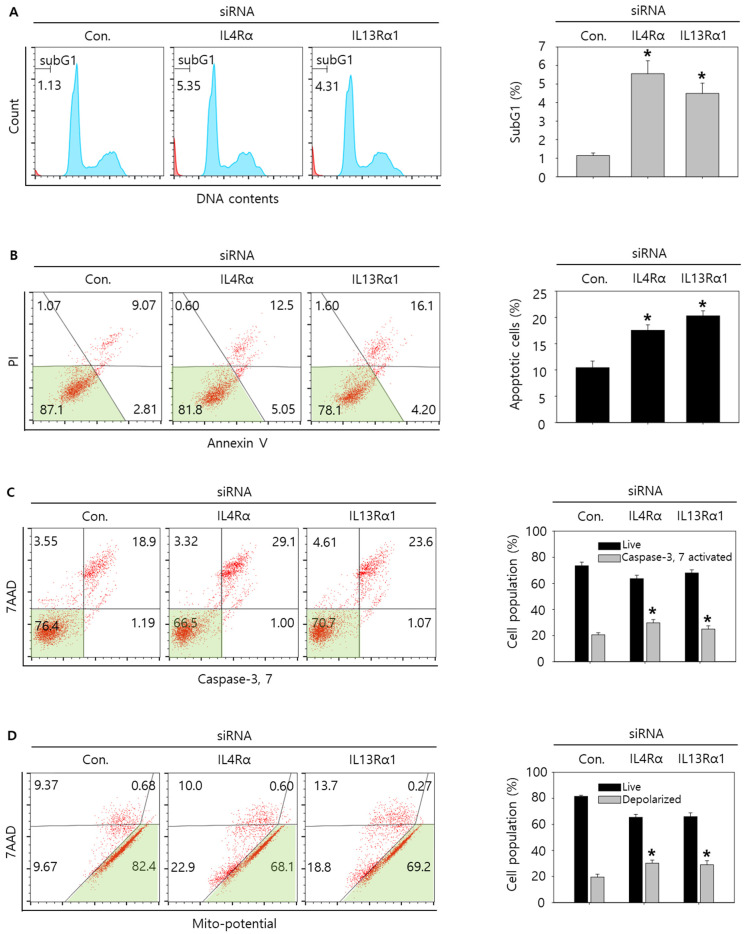
Cell cycle analysis and measurement of apoptosis by knockdown of IL4Rα or IL13Rα1 in SNU308 cells. (**A**) Cell cycle population was determined by Muse Cell Analyzer and Muse Cell Cycle Kit for 48 h after transfection of siRNA. (**B**) Annexin V staining was analyzed by Muse Cell Analyzer and Muse Annexin V and Dead Cell Kit for 48 h after transfection of siRNA. (**C**) Caspase-3 and -7 activities were measured by Muse Cell Analyzer and Muse Caspase-3/7 Kit for 48 h after transfection of siRNA. (**D**) Depolarization of mitochondria was detected by Muse Cell Analyzer and Muse Mito-Potential Kit for 48 h after transfection of siRNA. Quantitative graph was added to each result. * *p* < 0.05; asterisks mean a significant difference between control siRNA group and IL4Rα or IL13Rα1 siRNA group. Results are mean ± SD of at least three independent experiments.

**Figure 5:**
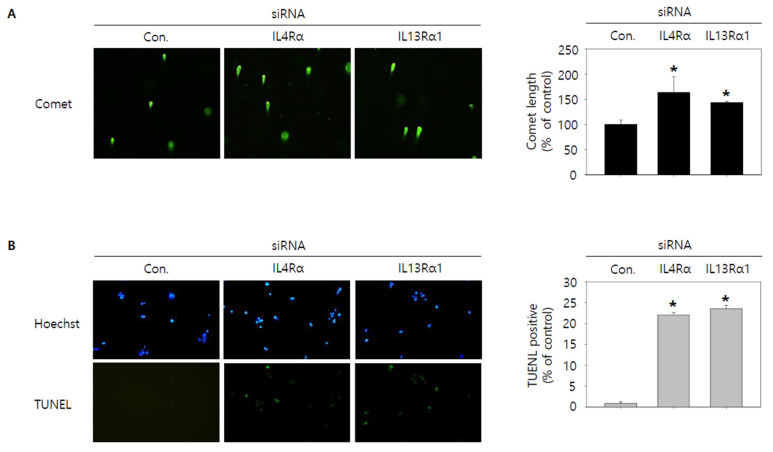
Visualization of DNA damage by knockdown of IL4Rα or IL13Rα1 in SNU308 cells. (**A**) Comet assay was conducted to visualize DNA damage in nucleus for 48 h after transfection of siRNA. (**B**) Blue color represents nucleus stained by Hoechst and green color represent TUNEL-positive nucleus for 48 h after transfection of siRNA. DNA damage of cell was observed by fluorescence microscopy. Green color in the cytogram means live cells. Quantitative graph was added to each result. * *p* < 0.05; asterisks mean a significant difference between control siRNA group and IL4Rα or IL13Rα1 siRNA group. Results are mean ± SD of at least three independent experiments.

**Figure 6:**
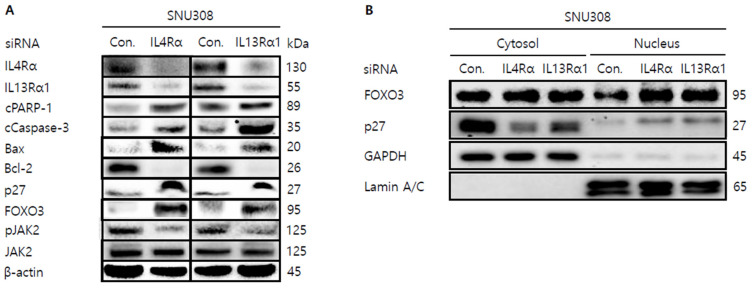
Western blotting analysis of proteins in SNU308 cells transfected with IL4Rα or IL13Rα1. (**A**) Total cell lysates Western blotting analysis in SNU308 for 48 h after transfection of siRNA. β-actin was used for a gel-loading control. (**B**) Nuclear fractional Western blotting analysis in SNU308 for 48 h after transfection of siRNA. GAPDH and Lamin A/C were used for a gel loading control for the cytosol and nuclear protein fractions, respectively. These results were representative images from three independent experiments.

Then, we tried to investigate whether knockdown of IL4Rα or IL13Rα1 induced apoptosis in SNU308 cells. The percentage of subG1 phase and total apoptotic cells (early and late apoptotic cells) increased after knockdown of IL4Rα or IL13Rα1 in SNU308 cells (Figure 4A,B). To verify the apoptotic effect of knockdown of IL4Rα or IL13Rα1, caspase-3 and -7 activity and mitochondria depolarization assay was performed. As shown in Figure 4C,D, assay results showed that knockdown of IL4Rα or IL13Rα1 caused the increase in the percentage of caspase-3 and -7 active and mitochondria-depolarized cells compared to the control siRNA. Comet assay results indicated that the comet length of nucleus in cells transfected with IL4Rα or IL13Rα1 was longer than that of the control siRNA. TUNEL-positive cells increased after knockdown of IL4Rα or IL13Rα1, which was associated with induction of DNA damage (Figure 5A,B). To examine the change in protein expression related to apoptosis and downstream signaling pathway under IL4Rα or IL13Rα1, Western blotting was performed (Figure 6A). We found that expression levels of cleaved PARP-1 (cPARP-1), cleaved caspase-3 (cCaspase-3), p27, Bax, and FOXO3 were increased but Bcl-2 and phosphorylated JAK2 (pJAK2) were decreased in IL4Rα or IL13Rα1 knockdown cells. Interestingly, either IL4Rα or IL13Rα1 knockdown caused downregulation of both of IL4Rα and IL13Rα1. Additionally, FOXO3 levels of nucleus increased in IL4Rα or IL13Rα1 knockdown cells, which caused the upregulation of p27 expression (Figure 6B). Taken together, our results demonstrated that knockdown of IL4Rα or IL13Rα1 might induce apoptosis in SNU308 cells via regulation of JAK2/FOXO3 pathways.

### 3.5. AZD1480 Displays Anti-Proliferative Activity in SNU308 Cells 

**Figure 7:**
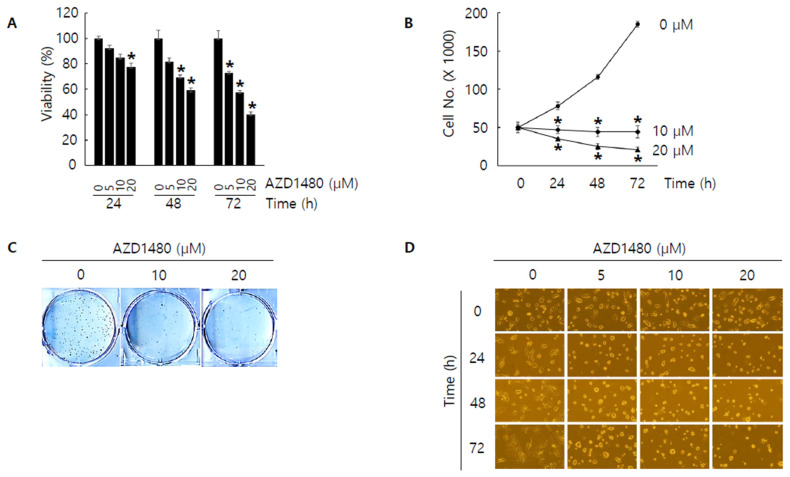
Anti-proliferative effect by AZD1480 treatment in SNU308 cells. (**A**) Cell viability (WST-1 assay) was measured for 24, 48, and 72 h after AZD1480 treatment. The percentage of SNU308 cell viability transfected with siRNAs against IL4Rα and IL13Rα1 was compared with the cells transfected with control siRNA (cell viability = 100%) at 24 h after transfection. (**B**) The number of viable cells was counted with hemocytometer for 24, 48, and 72 h after AZD1480 treatment. (**C**) After AZD1480 treatment, cells were grown for 14 days in fresh media containing 10% FBS. Crystal violet staining was performed to visualize colonies. (**D**) Cell morphological image was captured by microscopy for 24, 48, and 72 h after AZD1480 treatment. * *p* < 0.05; asterisks mean a significant difference between control group and treatment group. Results are mean ± SD of at least three independent experiments.

**Figure 8:**
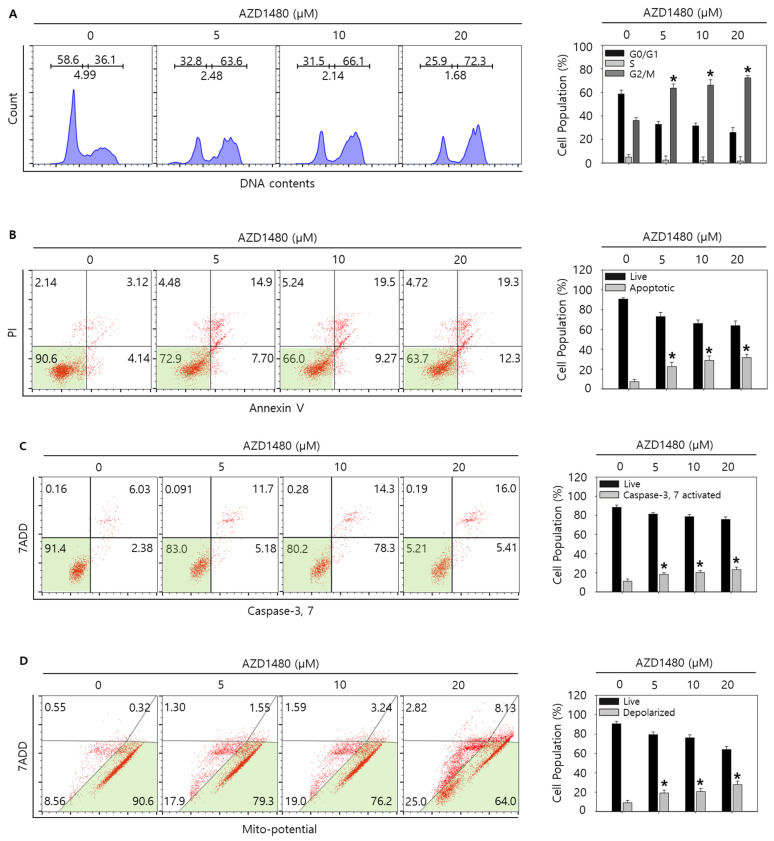
Cell cycle analysis and measurement of apoptosis by AZD1480 treatment in SNU308 cells. (**A**) Cell cycle population was determined by Muse Cell Analyzer and Muse Cell Cycle Kit for 48 h after AZD1480 treatment. (**B**) Annexin V staining was analyzed by Muse Cell Analyzer and Muse Annexin V and Dead Cell Kit for 48 h after AZD1480 treatment. (**C**) Caspase-3 and -7 activities were measured by Muse Cell Analyzer and Muse Caspase-3/7 Kit for 48 h after AZD1480 treatment. (**D**) Depolarization of mitochondria was detected by Muse Cell Analyzer and Muse Mito-Potential Kit for 48 h after AZD1480 treatment. Green color in the cytogram means live cells. Quantitative graph was added to each result. * *p* < 0.05; asterisks mean a significant difference between control group and treatment group. Results are mean ± SD of at least three independent experiments.

Since JAK2 was considered as one of the down-stream kinases under IL4Rα and IL13Rα1 complex and IL4Rα or IL13Rα1 knockdown decreased the expression of pJAK2 in SNU308 cells, we tried to evaluate the anti-proliferative effect of AZD1480, one of JAK2 inhibitors. After treatment of different concentrations of AZD1480, WST-1 assay was performed (Figure 7A). The result showed that viability decreased in dose- and time-dependent manners. Cell counting assay indicated that the number of living cells decreased significantly after treatment of AZD1480 (Figure 7B). Colony formation assay was conducted to visualize the anti-proliferative effect of AZD1480 in SNU308 cells (Figure 7C). We found that the number of colonies decreased significantly after treatment of AZD1480. Analysis of morphological cell images showed that cell density decreased and morphology was changed after AZD1480 treatment (Figure 7D). Cell cycle analysis results showed that the percentage of G2/M phase increased after AZD1480 treatment (Figure 8A). Overall, our results indicated that AZD1480 displayed anti-proliferative activity in SNU308 cells with dose and time dependent manner, which might be associated with G2/M cell cycle arrest.

### 3.6. AZD1480 Induces Apoptosis in SNU308 Cells via Regulation of JAK2/FOXO3 Pathway

**Figure 9:**
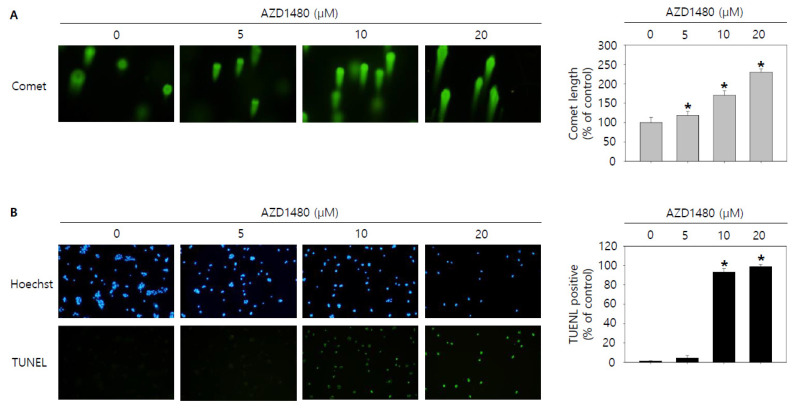
Visualization of DNA damage by AZD1480 treatment in SNU308 cells. (**A**) Comet assay was conducted to visualize DNA damage in nucleus for 48 h after AZD1480 treatment. (**B**) Blue color represents nucleus stained by Hoechst and green color represent TUNEL-positive nucleus for 48 h after AZD1480 treatment. DNA damage of cell was observed by fluorescence microscopy. Quantitative graph was added to each result. * *p* < 0.05; asterisks mean a significant difference between control group and treatment group. Results are mean ± SD of at least three independent experiments.

**Figure 10:**
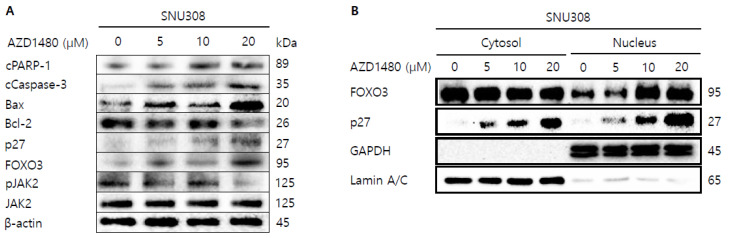
Western blotting analysis of proteins in SNU308 cells treated with by AZD1480. (**A**) Total cell lysates Western blotting analysis in SNU308 for 48 h after AZD1480 treatment. β-actin was used for a gel-loading control. (**B**) Nuclear fractional Western blotting analysis in SNU308 for 48 h after AZD1480 treatment. GAPDH and Lamin A/C were used for a gel loading control for the cytosol and nuclear protein fractions, respectively. These results were representative images from three independent experiments.

As shown in the result of 3.5, we found that AZD1480 exerted anti-proliferative effects on SNU308 cells. To evaluate the apoptotic effects of AZD1480 in SNU308 cells, we performed apoptosis assay using Annexin V and Dead Cell Kit, which showed that the percentage of total apoptotic cell rates (early and late apoptotic cells) increased after AZD1480 treatment in SNU308 cells (Figure 8B). We also measured the activity of caspase-3 and -7 which are typical indicator of apoptotic signaling pathway (Figure 8C). We found that the percentages of caspase-3 and -7 activated cells increased after AZD1480 treatment. In addition, we measured how much mitochondria were depolarized after treatment of AZD1480 in SNU308 cells (Figure 8D). We found that the percentage of depolarized SNU308 cells increased after AZD1480 cells. To visualize the DNA damage in SNU308 cells treated with AZD1480, TUNEL and Comet assays were performed. Comet assay was performed to measure the length of comet tail of DNA damage. As shown in Figure 9A, the length of comet tail increased after treatment of AZD1480 in SNU308 cells. TUNEL assay results showed that the percentage of TUNEL positive cells (indicator of DNA damage) increased after AZD1480 treatment (Figure 9B). Furthermore, we conducted Western blotting analysis to evaluate the signaling pathways related to apoptosis in SNU308 cells after AZD1480 treatment (Figure 10A). Our results indicated that expression levels of cPARP-1, cCaspase-3, p27, Bax, and FOXO3 were increased but Bcl-2 and pJAK2 were decreased in AZD1480 treated cells with dose dependent manner. Even though FOXO3 level of cytosol part was not changed, that of nucleus part increased in a dose-dependent manner and p27 expression levels of cytosol and nucleus part increased after AZD1480 treatment in SNU308 cells (Figure 10B). Taken together, our results suggests that AZD1480 induces apoptosis in SNU308 cells via regulation of JAK2/FOXO3 pathways.

## 4. Discussion

Even though gallbladder cancer is rare, it is the most common cancer in the biliary tract, accounting for more than 90% of biliary tract malignancy [30]. There are various therapeutic methods that have been known as the most effective for gallbladder cancer treatment such as surgical and radioactive treatment but prognosis of patients with gallbladder cancer still has not improved [31]. Thus, to overcome the limitations of the current therapeutic methods, alternative therapy should be developed. Recent studies showed that specific proteins in cells could play pivotal roles in cancer development and survival, which suggested that regulation of target protein could be an important clue of cancer treatment [32,33]. Our aim of this study is to find therapeutic targets for treatment of gallbladder cancer. We demonstrated that the downregulated IL4Rα or IL13Rα1 caused apoptosis in SNU308 gallbladder cells and investigated the anti-carcinogenic effect of AZD1480, a JAK2 inhibitor, in human gallbladder carcinoma SNU308 cells.

This study evaluated the expressions of IL4Rα and IL13Rα1 in human gallbladder carcinomas and presented IL4Rα/IL13Rα1 pathway is involved in the progression of gallbladder carcinomas. In univariate survival analysis, nuclear or cytoplasmic expressions of IL4Rα and IL13Rα1 were significantly associated with shorter OS and RFS of gallbladder carcinoma patients. The positivity of nuclear expression of IL4Rα was presented as an independent indicator of shorter survival gallbladder carcinoma patients. This prognostic significance of Nu-IL4Rα-positivity presents the expression pattern of IL4Rα and IL13Rα1 is used as prognostic indicators of gallbladder carcinoma patients. 

In survival analysis of gallbladder carcinoma patients, another interesting finding of this study is that nuclear localization of IL4Rα was presented as an independent indicator of shorter survival of gallbladder carcinoma patients. Considering IL4Rα and IL13Rα1 as cytokine receptor, the localization of IL4Rα and IL13Rα1 were expected on the plasma membrane. However, the expressions of IL4Rα and IL13Rα1 were dominant on cytoplasm and nuclei in gallbladder cancer cells. Consistently, nuclear expression of IL4Rα and IL13Rα1 was presented in soft tissue sarcomas, clear cell renal cell carcinoma [19], squamous cell carcinoma [34], and lung cancer [35]. Supportively, nuclear expression of IL4Rα and IL13Rα1 in human cancers was shown in a public database (The Human Protein Atlas; https://www.proteinatlas.org. date of last access: 03 January 2022) [36,37]. Furthermore, in a previous study evaluated for the prognostic significance of the expressions of IL4Rα and IL13Rα1 according to their subcellular localization, both nuclear expressions of IL4Rα and IL13Rα1 were the independent indicators of shorter OS and RFS of soft tissue sarcoma patients. This study also presented that nuclear expression of IL4Rα and IL13Rα1 was involved in pathogenesis important in the progression of gallbladder carcinomas. In this study, Nu-IL4Rα-positivity was significantly associated with higher tumor stage, presence of distant metastasis at diagnosis, and higher histologic grade. These results suggest nuclear localization of IL4Rα is important in the progression of gallbladder cancers.

ILs are a type of cytokine that mediates communication between cells, and regulates cell proliferation and differentiation [38]. It has been reported that there are various interleukins [38]. Among them, IL4 and IL13 are typical cytokines that are responsible for type II inflammatory response [7]. Recently, IL4 and IL13 have been considered not only target for inflammatory diseases but also the therapeutic target of cancer [18]. A recent study has shown that macrophages stimulated by IL4 and IL13 promoted invasion of breast cancer cells through Rho-GTPase regulation [39]. Moreover, clinical studies showed the safety and efficacy of several reagents targeting IL4 and IL13 receptors in pancreatic cancer [40]. Interestingly, inhibitory effects of IL4 and IL13 in human gallbladder cancer cells have hardly been investigated so far. To be best of our knowledge, this is the first study to demonstrate that IL4Rα and IL13Rα1 is involved in development of gallbladder cancer cells. Previously we reported that IL4Rα and IL13Rα1 (Type II receptor) is a prognostic marker and involved in the development of clear cell renal cell carcinoma (ccRCC) through JAK2/FOXO3 pathway [19]. Therefore, in this study, we tried to investigate the involvement of JAK2/FOXO3 pathway as one of down-stream signaling pathway in gallbladder cancer cells. Previously, Fu et al. reported that AG490, JAK2 tyrosine kinase inhibitor, suppressed the growth and invasiveness of GBC-SD and SGC-996 gallbladder cancer cells via inhibition of JAK2 and activation p53 [41].

JAK2 is tyrosine kinase that plays crucial roles in cell growth and cell division [42]. Interestingly, misregulation and mutation of JAK2 were observed frequently in many myeloproliferative diseases and various cancers, which suggested that JAK2 could be an important target for cancer treatment [43,44]. Many studies demonstrated the role of JAK2 in various cancers. Inhibition of JAK2 signaling pathway suppressed the proliferation of gastric cancer in vitro and in vivo [45]. This study demonstrated that JAK2/STAT3 signaling pathway regulated various cytokines and growth factors that play an important role in gastric cancer cell growth [45]. Furthermore, it was recently reported that inhibition of JAK2 signaling pathway induced apoptosis in colorectal cancer cells [46]. This study elucidated that the efficacy of anti-cancer reagent was mediated by JAK2/STAT3 signaling pathway in colorectal cancer HCT116 and SW480 cells [46]. A previous study investigated the role of JAK2 signaling pathway in human bladder cancer E-J and 5637 cells [47]. However, the effect of AZD1480, a JAK2 inhibitor, in human gallbladder cancer has hardly been investigated. We showed that AZD1480 induced apoptosis via regulation of JAK2/FOXO3 pathway in human gallbladder cancer SNU308 cells. Since we observed the anti-cancer activity of AZD1480 in SNU308 cells, we are planning to develop novel small chemical drugs from synthetic and FDA approved drug library to target JAK2.

Transcription factor forkhead Box O3 (FOXO3) is related to various tumor development process such as metastasis and angiogenesis [48]. We found that AZD1480 treatment increased expression level of FOXO3, especially in the nucleus part of cell. Furthermore, knockdown of IL4Rα or IL13Rα1 increased expression level of FOXO3 in SNU308 cells. Interestingly, knockdown of IL4Rα or IL13Rα1 downregulated the phosphorylated JAK2 expression level. Our results suggested that FOXO3 signaling seemed to be mediated by JAK2 signaling pathway and regulation of JAK2/FOXO3 signaling pathway suppressed cell proliferation and induced apoptosis in SNU308 cells. Since FOXO3 acts as a transcription factor and it plays a crucial role in cell survival and proliferation, FOXO3 has been recognized as an important therapeutic target for cancer treatment [49,50]. Therefore, it is crucial to investigate the role of FOXO3 in various cancer cells. Several studies investigated the relationship between FOXO3 and gallbladder cancers. A recent study has shown that activation of FOXO3 by polyphyllin I induced apoptosis in human gallbladder cancer cells [51]. Another study demonstrated that ratio of phosphorylated and total FOXO3 could be an important prognostic marker of tumor recurrence in patients with gallbladder cancer [52]. Additionally, it was reported that FOXO3 expression could play a pivotal role in prognosis of gallbladder cancer [53].

## 5. Conclusions

In conclusion, this study showed the expression of IL4Rα and IL13Rα1 was used as a potential prognostic marker of gallbladder carcinomas and presented nuclear expression of IL4Rα as an independent indicator of shorter OS and FRS. By investigating a mechanism for the relationship between IL4R and IL13R1 expressions and cell survival, we demonstrated that knockdown of IL4Rα or IL13Rα1 through siRNA suppressed cell proliferation induced apoptosis in SNU308 cells via regulation of JAK2/FOXO3 pathway, which was verified by using AZD1480, a JAK2 inhibitor, to SNU308 cells. Therefore, our findings suggests that the IL4Rα and IL13Rα1 complex and JAK2 signaling pathway could be efficient therapeutic targets for gallbladder cancer treatment.

## Figures and Tables

**Table 1:** Clinical variables and the expression of IL4Rα and IL13Rα1 in gallbladder carcinomas.

Characteristics		No.	Nu-IL4Rα		Cy-IL4Rα		Nu-IL13Rα1		Cy-IL13Rα1	
			Positive	*p*	Positive	*p*	Positive	*p*	Positive	*p*
Age (years)	<65 y	60	31 (52%)	0.010	36 (60%)	0.003	46 (77%)	0.921	28 (47%)	0.208
	≥65 y	62	46 (74%)		52 (84%)		48 (77%)		36 (58%)	
Sex	Male	62	42 (68%)	0.282	51 (82%)	0.011	41 (66%)	0.004	33 (53%)	0.863
	Female	60	35 (58%)		37 (62%)		53 (88%)		31 (52%)	
CEA	Normal	98	60 (61%)	0.195	70 (71%)	0.658	74 (76%)	0.594	52 (53%)	0.651
	Elevated	21	16 (76%)		16 (76%)		17 (81%)		10 (48%)	
CA19-9	Normal	79	49 (62%)	0.622	57 (72%)	0.968	62 (78%)	0.616	41 (52%)	0.644
	Elevated	39	26 (67%)		28 (72%)		29 (74%)		22 (56%)	
TNM stage	I and II	75	42 (56%)	0.04	53 (71%)	0.649	59 (79%)	0.591	40 (53%)	0.807
	III and IV	47	35 (74%)		35 (74%)		35 (74%)		24 (51%)	
T category	T1	32	16 (50%)	0.035	21 (66%)	0.595	21 (66%)	0.214	12 (38%)	0.219
	T2	59	35 (59%)		42 (71%)		50 (85%)		34 (58%)	
	T3	27	23 (85%)		22 (81%)		20 (74%)		15 (56%)	
	T4	4	3 (75%)		3 (75%)		3 (75%)		3 (75%)	
LN metastasis	Absence	93	58 (62%)	0.759	69 (74%)	0.363	73 (78%)	0.497	50 954%)	0.605
	Presence	29	19 (66%)		16 (55%)		21 (72%)		14 (48%)	
Distant metastasis	Absence	114	69 (61%)	0.025	80 (70%)	0.069	88 (77%)	0.887	60 (53%)	0.885
	Presence	8	8 (100%)		8 (100%)		6 (75%)		4 (50%)	
Histologic type	Adenocarcinoma NOS	118	73 (62%)	0.299	84 (71%)	0.450	92 (78%)	0.166	62 (53%)	0.510
	Adenosquamous carcinoma	3	3 (100%)		3 (100%)		2 (67%)		2 (67%)	
	Squamous cell carcinoma NOS	1	1 (100%)		1 (100%)		0 (0%)		0 (0%)	
Histologic grade	Low	50	25 (50%)	0.012	36 (72%)	0.979	35 (70%)	0.123	19 (38%)	0.008
	High	72	52 (72%)		52 (72%)		59 (82%)		45 (63%)	
Cy-IL13Rα1	Negative	58	29 (50%)	0.004	34 (59%)	0.002	33 (57%)	<0.001		
	Positive	64	48 (75%)		54 (84%)		61 (95%)			
Nu-IL13Rα1	Negative	28	12 (43%)	0.011	19 (68%)	0.566				
	Positive	94	65 (69%)		69 (73%)					
Cy-IL4Rα	Negative	34	13 (38%)	<0.001						
	Positive	88	64 (73%)							

Abbreviations: Nu-IL4Rα; nuclear expression of IL4Rα, Cy-IL4Rα; cytoplasmic expression of IL4Rα, Nu-IL13Rα1; nuclear expression of IL13Rα1, Cy-IL13Rα1; cytoplasmic expression of IL13Rα1.

**Table 2:** Univariate analysis of overall survival and relapse-free survival in gallbladder carcinoma patients.

Characteristics	No.	OS			RFS		
		HR	95% CI	*p*	HR	95% CI	*p*
Age, y ≥ 65 (vs. <65)	62/122	2.603	1.625–4.168	<0.001	2.438	1.539–3.863	<0.001
Sex, female (vs. male)	60/122	0.736	0.471–1.150	0.178	0.71	0.456–1.103	0.127
CEA, elevated (vs. normal)	21/119	1.487	0.856–2.584	0.159	1.394	0.804–2.416	0.237
CA19-9, elevated (vs. normal)	39/118	1.729	1.091–2.741	0.020	1.653	1.047–2.610	0.031
TNM stage, I and II (vs. III and IV)	47/122	3.658	2.318–5.773	<0.001	3.21	2.045–5.039	<0.001
T category, T1	32/122	1		<0.001	1		<0.001
T2	59/122	2.511	1.279–4.929	0.007	2.716	1.388–5.313	0.004
T3	27/122	9.948	4.813–20.563	<0.001	9.115	4.404–18.866	<0.001
T4	4/122	10.679	3.283–34.738	<0.001	11.195	3.436–36.482	<0.001
LN metastasis, presence (vs. absence)	29/122	1.978	1.217–3.215	0.006	1.857	1.145–3.011	0.012
Distant metastasis, presence (vs. absence)	8/122	6.288	2.849–13.878	<0.001	5.039	2.301–11.034	<0.001
Histologic type, adenocarcinoma NOS	118/122	1		0.002	1		0.005
Adenosquamous carcinoma	3/122	4.416	1.372–14.209	0.013	3.635	1.133–11.665	0.030
Squamous cell carcinoma NOS	1/122	15.704	1.960–125.812	0.009	13.854	1.752–109.584	0.013
Histologic grade, high (vs. low)	72/122	2.874	1.747–4.727	<0.001	2.8	1.718–4.564	<0.001
Cy-IL13Rα1, positive (vs. negative)	64/122	1.987	1.250–3.161	0.004	2.008	1.271–3.172	0.003
Nu-IL13Rα1, positive (vs. negative)	94/122	2.197	1.159–4.168	0.016	2.31	1.220–4.376	0.010
Cy-IL4Rα, positive (vs. negative)	88/122	1.895	1.092–3.288	0.023	2.048	1.183–3.548	0.011
Nu-IL4Rα, positive (vs. negative)	77/122	4.614	2.575–8.269	<0.001	4.019	2.311–6.988	<0.001

Abbreviations: OS, overall survival; RFS, relapse-free survival; HR, hazard ratio; 95% CI, 95% confidence interval, Nu-IL4Rα; nuclear expression of IL4Rα, Cy-IL4Rα; cytoplasmic expression of IL4Rα, Nu-IL13Rα1; nuclear expression of IL13Rα1, Cy-IL13Rα1; cytoplasmic expression of IL13Rα1.

**Table 3:** Multivariate analysis of overall survival and relapse-free survival in gallbladder carcinoma patients.

Characteristics	OS			RFS		
	HR	95% CI	*p*	HR	95% CI	*p*
Age, y ≥ 65 (vs. <65)	2.755	1.675–4.533	<0.001	2.646	1.633–4.287	<0.001
TNM stage, I and II (vs. III and IV)	2.605	1.260–5.386	0.010	2.098	1.023–4.300	0.043
T category, T1	1		0.048	1		0.016
T2	2.202	1.065–4.552	0.033	2.502	1.218–5.138	0.012
T3	3.264	1.179–9.036	0.023	3.805	1.383–10.471	0.010
T4	6.302	1.602–24.788	0.008	8.146	2.063–32.156	0.003
Nu-IL4Rα, positive (vs. negative)	3.379	1.825–6.254	<0.001	2.919	1.622–5.253	<0.001

Abbreviations: OS, overall survival; RFS, relapse-free survival; HR, hazard ratio; 95% CI, 95% confidence interval, Nu-IL4Rα; nuclear expression of IL4Rα.

## Data Availability

The datasets used in the current study are available from the corresponding author upon reasonable request.
